# Destruction of an Insane Asylum

**Published:** 1861-01

**Authors:** 


DESTRUCTION OF AN INSANE ASYLUM.—The Western Insane Asylum at Hopkinsville, Kentucky, erected at a cost of upwards of $200,000, was totally destroyed by fire early in December. All the inmates, with one exception, were saved. Had the building been protected with a metallic roof the accident would not have occurred.

